# Deep Phenotyping Reveals Distinct Immune Signatures Correlating with Prognostication, Treatment Responses, and MRD Status in Multiple Myeloma

**DOI:** 10.3390/cancers12113245

**Published:** 2020-11-04

**Authors:** Konstantinos Papadimitriou, Nikolaos Tsakirakis, Panagiotis Malandrakis, Panagiotis Vitsos, Andreas Metousis, Nikolaos Orologas-Stavrou, Ioannis Ntanasis-Stathopoulos, Nikolaos Kanellias, Evangelos Eleutherakis-Papaiakovou, Panagiotis Pothos, Despina Fotiou, Maria Gavriatopoulou, Efstathios Kastritis, Meletios-Athanasios Dimopoulos, Evangelos Terpos, Ourania E. Tsitsilonis, Ioannis V. Kostopoulos

**Affiliations:** 1 Department of Biology, School of Sciences, National and Kapodistrian University of Athens, 15784 Athens, Greece; kostpap@med.uoa.gr (K.P.); nicktsak@biol.uoa.gr (N.T.); pvitsos@biol.uoa.gr (P.V.); andreas.metousis@stud.ki.se (A.M.); norologas@med.uoa.gr (N.O.-S.); ppothos@biol.uoi.gr (P.P.); 2 Department of Clinical Therapeutics, School of Medicine, National and Kapodistrian University of Athens, 11528 Athens, Greece; panosmalan@med.uoa.gr (P.M.); johnntanasis@med.uoa.gr (I.N.-S.); nkanellias@med.uoa.gr (N.K.); evelepapa@med.uoa.gr (E.E.-P.); desfotiou@med.uoa.gr (D.F.); mgavria@med.uoa.gr (M.G.); ekastritis@med.uoa.gr (E.K.); mdimop@med.uoa.gr (M.-A.D.); eterpos@med.uoa.gr (E.T.)

**Keywords:** multiple myeloma, bone marrow microenvironment, immune profiling, immune signatures, minimal residual disease

## Abstract

**Simple Summary:**

In Multiple Myeloma (MM) malignant cells accumulate in the bone marrow (BM), where they interact with various cell populations. These complex interactions impose mechanisms of tumor growth and proliferation, immune surveillance and immune evasion. The aim of the present study was a detailed immune characterization of MM during the course of the disease, in order to highlight signatures which are clinically relevant. Analyses of both BM and peripheral blood (PB) in matched patients’ samples, we showed that PB cannot representatively reflect the BM microenvironment. Particular immune signatures in BM and PB significantly correlated with established prognostic features and could independently associate with distinct responses to the same induction therapy. Moreover, our data provide evidence of a diverse immune profile according to patients’ MRD status post treatment. Finally, we provide insights that unique PB immune profiles may be used for the prediction of MRD status through a simple non-invasive approach.

**Abstract:**

Despite recent advances, Multiple Myeloma (MM) remains an incurable disease with apparent heterogeneity that may explain patients’ variable clinical outcomes. While the phenotypic, (epi)genetic, and molecular characteristics of myeloma cells have been thoroughly examined, there is limited information regarding the role of the bone marrow (BM) microenvironment in the natural history of the disease. In the present study, we performed deep phenotyping of 32 distinct immune cell subsets in a cohort of 94 MM patients to reveal unique immune profiles in both BM and peripheral blood (PB) that characterize distinct prognostic groups, responses to induction treatment, and minimal residual disease (MRD) status. Our data show that PB cells do not reflect the BM microenvironment and that the two sites should be studied independently. Adverse ISS stage and high-risk cytogenetics were correlated with distinct immune profiles; most importantly, BM signatures comprised decreased tumor-associated macrophages (TAMs) and erythroblasts, whereas the unique Treg signatures in PB could discriminate those patients achieving complete remission after VRd induction therapy. Moreover, MRD negative status was correlated with a more experienced CD4- and CD8-mediated immunity phenotype in both BM and PB, thus highlighting a critical role of by-stander cells linked to MRD biology.

## 1. Introduction

Multiple Myeloma (MM) is a heterogeneous neoplastic disorder characterized by a multi-level variety of clinical symptoms, cell phenotypes, (cyto)genetic and epigenetic background, and clonal evolutionary patterns. Much effort has been made for the identification of well-defined criteria to stratify patients into distinct prognostic groups. Current stratification criteria have been proven useful in the daily clinical practice; however, they still have specific limitations, evidenced by the fact that even among patients within the same prognostic group there is significant heterogeneity in outcomes even with the same therapeutic approach [1,2].

MM is an incurable hematological malignancy as, despite temporary achievement of deep responses, the majority of patients will eventually relapse. Research efforts have mainly focused on illustrating the biologic features of clonal plasma cells and therapies have traditionally aimed to directly target the malignant population and/or deregulate functions or pathways crucial for clonal cell survival and expansion [3,4]. However, the effective utilization of immunomodulatory drugs (IMiDs) in the clinical setting coincided the beginning of a major shift in understanding the underlying mechanisms of effective anti-cancer treatments, which comprise a three-pronged approach: (i) induction of direct tumor cell apoptosis; (ii) interference in tumor cell–microenvironment interactions; and (iii) enhancement of the anti-tumor immune response [5,6]. IMiDs act pleiotropically, exhibiting immunomodulatory, anti-angiogenic, anti-inflammatory, and anti-proliferative properties, and likely alter the bone marrow (BM) microenvironment. However, the relative contribution of each parameter in IMiDs anti-myeloma activity is still unclear, and further complicated by the highly heterogeneous IMiDs’ efficacy in MM patients [5,6,7,8,9,10].

Myeloma cells grow and proliferate in the BM, a niche comprising numerous and diverge cell subsets. There is now sufficient evidence of a constant and dynamic interplay between myeloma cells and by-stander BM cell subsets, applying both on the supportive role of the latter in the survival and proliferation of malignant cells, but also on the balance between the host’s anti-tumor immune responses and the immune-escape mechanisms developed by myeloma cells [11,12,13]. This complicated matrix of interactions constitutes a real challenge in unveiling the involvement of the BM microenvironment in the natural history of the disease [14,15]. In the present study, we applied deep phenotype analysis to characterize the immune profile of peripheral blood (PB) and BM cells at different disease stages, and correlated PB and BM niche signatures with the clinical course of MM.

## 2. Patients and Methods 

### 2.1. Patients

The cohort analyzed comprised 15 smoldering MM (sMM), 8 plasma cell leukemias (PCL), and 94 MM patients (*n* = 53 at the time of diagnosis; *n* = 51 at minimal residual disease (MRD) evaluation, including 10 patients evaluated at both time-points), who were treated and followed at the Department of Clinical Therapeutics of the National and Kapodistrian University of Athens. The protocol was approved by the local ethics committee (Prot. No 116/28 February 2018). Prior to sampling, all patients were informed of the purposes of the study and signed an informed consent according to the Declaration of Helsinki. 

To eliminate treatment effect alterations in immune profiling and evaluate the clear effect of immune cell distribution during the clinical course of the disease, the cohort of newly-diagnosed MM patients (NDMM; *n* = 53) was homogenously treated receiving bortezomib, lenalidomide, and dexamethasone (VRd) as induction therapy. Accordingly, all patients evaluated for MRD (*n* = 51), achieved complete remission (CR) after receiving VRd followed by high-dose therapy and autologous stem cell transplantation (ASCT). BM aspirates and/or PB samples were drawn from all patients, including 51 patients (26 NDMM and 25 patients at the time of MRD evaluation) who provided both BM and PB matched samples. The clinical characteristics of patients are presented in Table 1. All samples were assessed with Next-generation flow cytometry (NGF) panels for MRD detection [16]; 155 samples were analyzed for T cells and their subpopulations; 84 samples were analyzed for myeloid-derived suppressor cells (MDSCs) and their subsets.

### 2.2. Next-Generation Flow Cytometry

Diagnostic and MRD samples were analyzed with the NGF protocol according to the EuroFlow guidelines [16]. The NGF 8-color antibody panels applied in lysed PB and BM samples consisted of tube 1: CD38-FITC, CD56-PE, CD45-PerCPCy5.5, CD138-BV421, CD27-BV510, CD19-PC7, CD117-APC, CD81-APCC750; and tube 2: CD38-FITC, CD56-PE, CD45-PerCPCy5.5, CD138-BV421, CD27-BV510, CD19-PC7, Kappa-APC, Lambda-APCC750. Samples were analyzed for aberrant plasma cell (APC) discrimination from their normal plasma cell counterpart and for subset distribution (Table 2). MRD positivity was defined when more than 20 clonal APCs were detected at minimum number of 10 × 10^6^ events recorded per patient sample. BD FACSCantoII (BD Bioscience, San Jose, CA, USA) was used for sample acquisition and analysis was conducted with the Infinicyt software (Cytognos S.L., Salamanca, Spain).

### 2.3. Immune Profiling

Immune profiling was performed utilizing three 8-color antibody combinations, designed to characterize T cell and MDSC subsets. For T cell characterization, the two panels comprised the markers: CD3-FITC, CD4-APC-Cy7, CD8-PerCPCy5.5, CD25-APC, FoxP3-PE, CD127-BV510, CD39-BV421, Ki67-BV510, CD45RA-PC7, CD45RO-PerCPCy5.5, CTLA4-BV421, HLA-DR-PC7. The MDSC panel was designed for discriminating the various MDSC subsets and M1/M2 monocytes and comprised the markers: CD14-FITC, CD11b-PE, 7-AAD, CD124-BV421, CD33-BV510, HLA-DR-PC7, CD15-APC, lin (CD3, CD19, CD56)-APC-Cy7. Antibody clones and providers are listed in Table S1. Both T cell panels were applied on whole lysed PB and BM samples; for the MDSC panel, mononuclear cells were isolated after Ficoll density gradient centrifugation. Acquisition was performed on BD FACSCantoII and FlowJo (BD, Franklin Lakes, NJ, USA) was used for data analysis (gating strategy presented in Figure S1A–C). The detailed list of immune subsets analyzed and their phenotypes is presented in Table 2.

### 2.4. Cytogenetics

Cytogenetic analysis was performed by interphase fluorescence in-situ hybridization (i-FISH) for the most common aberrations detected in MM and particularly for t(4;14), t(11;14), t(14;16), del(13q)/monosomy 13, del(17p13), and add(1q21). Patients with at least one aberration of t(4;14), t(14;16), del(17p13), or add(1q21) were considered as high-risk (HR), whereas absence of these abnormalities was a sign of low-risk (LR) prognostication. Commercially available probes (Abbott Molecular, III, USA) were applied on the purified plasma cell population following an established protocol described in detail elsewhere [17,18].

### 2.5. Statistical Analysis

Quantitative variables were described by measures of central tendency (mean, median) and dispersion (SD) and were analyzed with the appropriate parametric and non-parametric models (*t*-test/Mann–Whitney U test for two group comparisons, one-way Anova/Kruskal Wallis for three group comparisons) to examine for differences among groups. The distribution of measures in each group was tested with Kolmogorov–Smirnoff normality test; paired *t*-test or Wilcoxon test were used to compare parametric and nonparametric data from matched BM/PB samples, accordingly. To evaluate the differences between BM and PB for the whole immune pattern, we used the Friedman non-parametric test. Multiple logistic regression was used for the prediction of positive MRD probability. Principal component analysis (PCA) was performed with Clustvis, a web tool for visualizing clustering of multivariate data [19]. Statistical analysis was performed with SPSS V25.0 (IBM, Armonk, NY, USA). A *p* value of <0.05 was considered statistically significant (* *p* < 0.05; ** *p* < 0.01; *** *p* < 0.001).

## 3. Results

### 3.1. Composition of the BM Microenvironment at Different Stages of MM Progression

**Figure 1:**
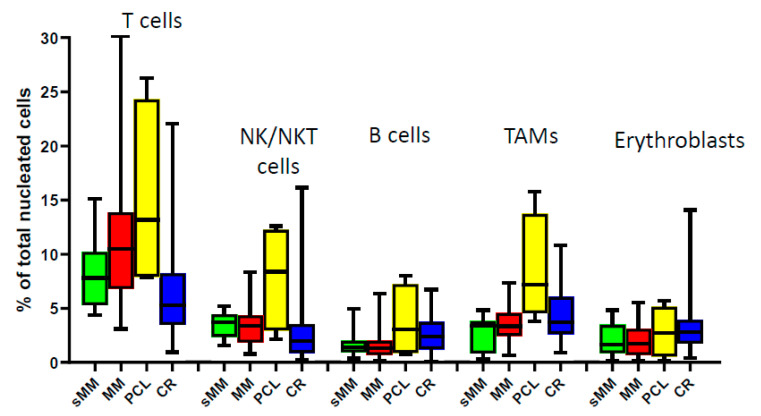
Bone marrow composition of major immune subsets at different stages during MM progression. sMM: smoldering myeloma; MM: multiple myeloma; PCL: plasma cell leukemia; CR: complete remission; NK/NKT: natural killer cells/natural killer T cells; TAMs: tumor-associated macrophages.

The comparison of the relative frequencies of the major immune subsets (T, NK/NKT, B cells, tumor-associated macrophages (TAMs), and erythroblasts) in the BM of patients at different, although related, plasma cell dyscrasias (sMM, MM, PCL) and at the time of CR after treatment did not show any statistically significant differences, mainly due to the apparent heterogeneity of each subset’s distribution among patients’ samples (Figure 1). The prevalence of T cells showed a gradual increase during progression, but this was not observed for the other subsets. In patients with PCL, we observed a unique profile with higher percentages of all analyzed subsets compared to sMM and MM, as circulating APCs in PCL are less dependent from the BM niche [20]. The variability of the BM microenvironmental composition among patients indicates the apparent heterogeneity of MM beyond the molecular level, thus importing an additional challenge for efficient patients’ stratification.

### 3.2. Peripheral Blood Cannot Reflect the Bone Marrow Microenvironment

We further investigated whether analysis of PB could resonate the BM microenvironment, thus reducing the need for invasive sampling. Therefore, we performed paired analysis of different immune subsets from matched BM and PB samples of MM patients both at diagnosis (*n* = 26) and at CR (*n* = 25).

**Figure 2:**
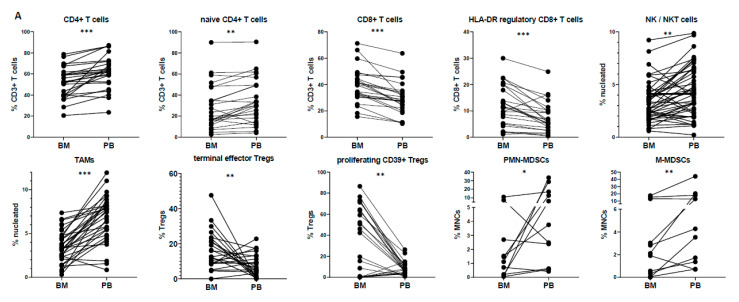

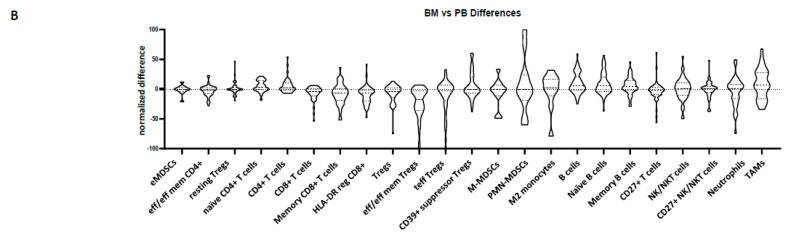
(**A**) Pairwise comparison of subset distributions between the bone marrow (BM) and peripheral blood (PB); (**B**) differences plot displaying the pairwise differences (PB minus BM) of normalized expression measures for all immune subsets. Subsets that tend to mirror between PB and BM appear on the left side of the plot. * *p* < 0.05; ** *p* < 0.01; *** *p* < 0.001.

At diagnosis, certain populations showed statistically significant differences between BM and PB (Figure 2). In particular, the mean prevalence of CD4+ T cells among CD3+ T cells was more abundant in PB (61.4%) than in the BM (51.1%; *p* < 0.001), mainly due to the relative increase of the naïve CD4+ T subset (34.3% in PB vs. 28.4% in BM; *p* < 0.01). The CD8+ T cell compartment was higher in the BM (36.8% in PB vs. 46.5% in BM; *p* < 0.001), due to the apparent increase of the HLA-DR regulatory CD8+ T subset (7.3% in PB vs. 11.9% in BM; *p* < 0.001). The prevalence of NK/NKT cells and monocytes/TAMs was higher in PB (NK/NKT cells: 4.4% in PB vs. 3.2% in BM; *p* < 0.01; monocytes/TAMs: 6.6% in PB vs. 3.1% in BM; *p* < 0.001), whereas no specific differences were observed regarding the total percentages of Tregs and MDSCs (Table S2). Nevertheless, subset analysis of Tregs and MDSCs revealed higher frequencies of effector/effector memory Tregs in the BM (9.3% in PB vs. 14.6% in BM; *p* < 0.05), as well as terminal effector Tregs (7.4% in PB vs. 16.0% in BM; *p* = 0.01) and proliferating CD39+ suppressor Tregs (8.1% in PB vs. 4.5% in BM; *p* < 0.01). Lastly, PMN-MDSCs and M-MDSCs were increased in PB vs. BM (*p* < 0.05 and *p* < 0.01, respectively), when evaluated in the total number of MDSCs (Figure 2A).

These discrepancies between the different compartments (PB vs. BM) were seen only at the time of MM diagnosis; the only differences maintained at CR were those of total CD4+ and CD8+ T cell compartments among CD3+ T cells (for CD4: 46.3% in PB vs. 40.9% in BM; *p* < 0.001; for CD8: 43.8% in PB vs. 53.1% in BM; *p* < 0.001). Interestingly, the prevalence of proliferating naïve CD4+ T cells showed a significant increase in PB vs. BM at diagnosis (0.9% in PB vs. 0.6% in BM; *p* = 0.014), but the opposite divergence at CR (0.2% in PB vs. 0.3% in BM; *p* = 0.006). The immune pattern for all subsets in BM vs. PB at both diagnosis and CR is depicted in detail in Table S2. 

Overall, if one considers the wide immune profile analyzed, the above-mentioned differences (at diagnosis and/or CR) between the two sites were only marginal. The vast majority of subsets showed high heterogeneity among patients and sites, hence not highlighting clear dissimilarities between BM and PB. In the same context, the absence of differences does not imply direct mirroring due to this variability; only a few subsets in PB mirrored their counterparts in BM, with minimum difference values as measured by SD (resting Tregs, eMDSCs, effector/effector memory CD4+ T cells) (Figure 2B).

### 3.3. Immune Profiling May Differ in Separate Prognostic Groups

We next tried to evaluate whether the various immune subsets at diagnosis showed any differential distribution among the different prognostic categories. Due to the apparent variability among compartments, immune distribution in BM and PB were examined separately.

**Figure 3:**
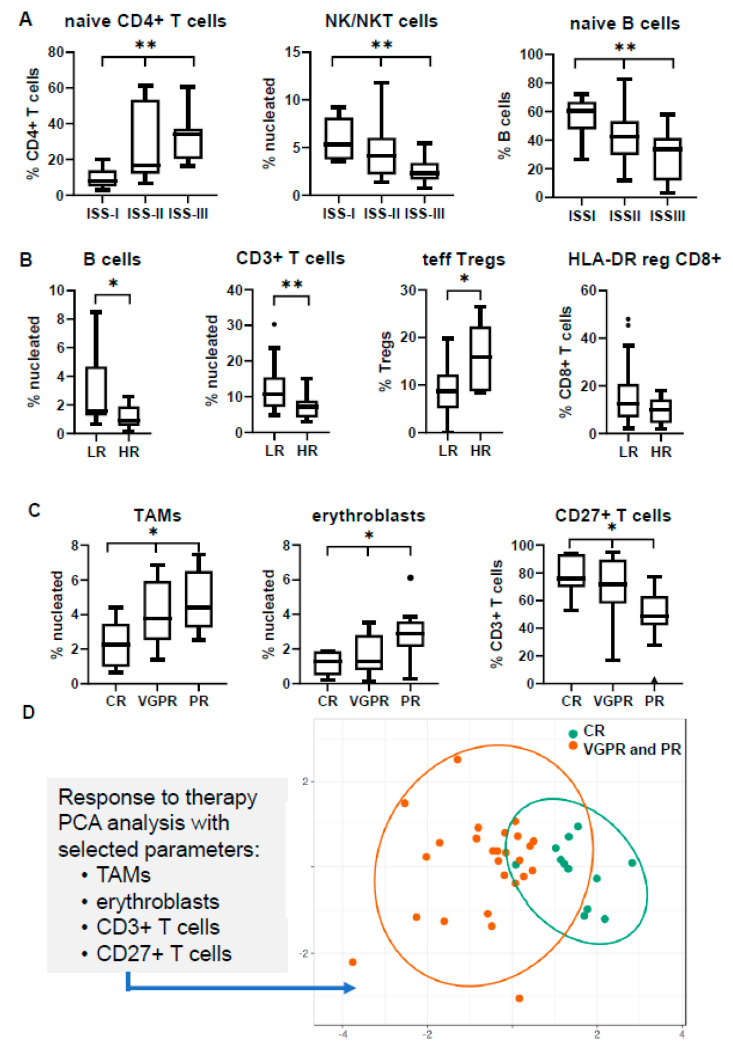
Distribution of bone marrow (BM) subsets in different clinical and prognostic groups at the time of diagnosis. Comparisons refer to (**A**) the International Staging System (ISS); (**B**) cytogenetics; (**C**) response to therapy; (**D**) PCA analysis of selected BM subsets according to therapeutic response. * *p* < 0.05, ** *p* < 0.01; HR: high-risk; LR: low-risk; CR: complete response; VGPR: very good partial response; PR: partial response; TAMs: tumor-associated macrophages; PCA: principal component analysis.

**Figure 4:**
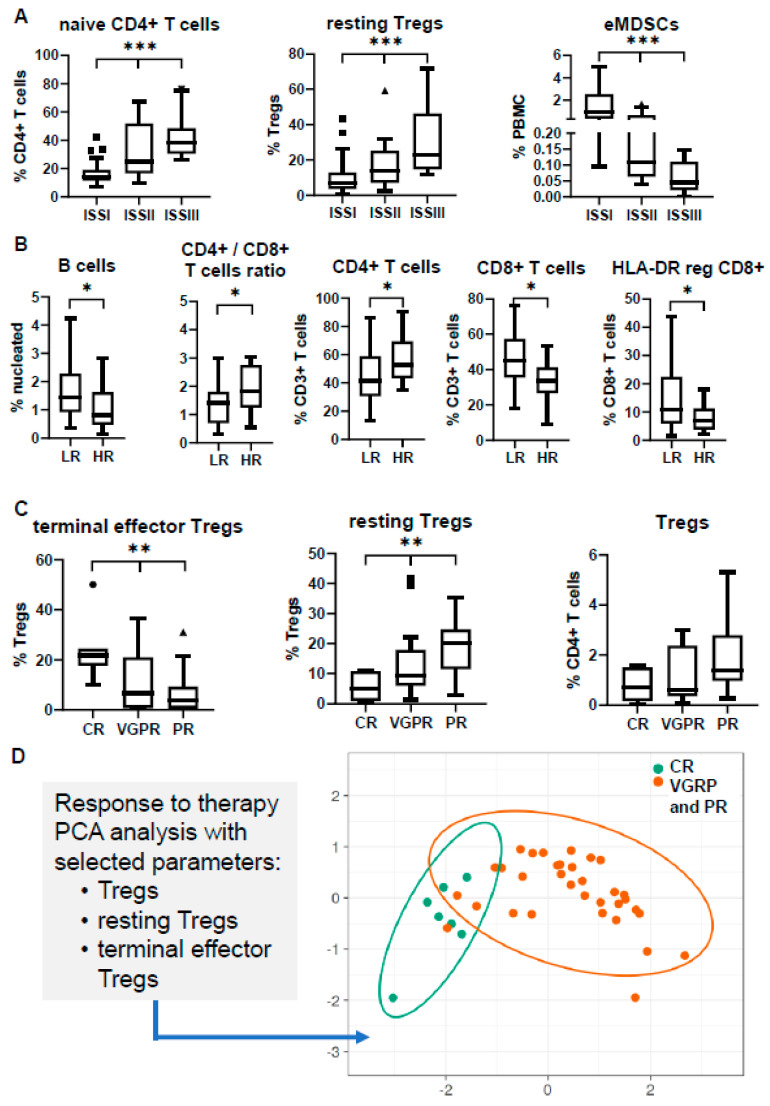
Peripheral blood (PB) profiling of different clinical and prognostic groups at the time of diagnosis. Comparisons refer to (**A**) International Staging System (ISS); (**B**) cytogenetics; (**C**) response to therapy; (**D**) PCA analysis of selected PB subsets according to therapeutic response. * *p* < 0.05; ** *p* < 0.01; *** *p* < 0.001; MDSCs: myeloid-derived suppressor cells; HR: high-risk; LR: low-risk; CR: complete response; VGPR: very good partial response; PR: partial response; PCA: principal component analysis.

Significant differences were observed in various subsets among the different ISS stages. The most obvious discrepancy was revealed in the relative abundance of naïve CD4+ T cells, showing a gradual increase towards the most adverse prognostic groups, with the same trend for both BM and PB (median values in BM: 7.9% in ISS-I vs. 16.8% in ISS-II vs. 34.0% in ISS-III; *p* = 0.004; median values in PB: 13.9% in ISS-I vs. 24.9% in ISS-II vs. 38.1% in ISS-III; *p* < 0.001). In BM, a gradual decrease towards ISS-III stage was also prominent for the NK/NKT subset (median: 5.3% in ISS-I vs. 4.1% in ISS-II vs. 2.3% in ISS-III; *p* = 0.006) and the relative frequency of naïve B cells among total B cells (median: 60.6% in ISS-I vs. 42.3% in ISS-II vs. 33.7% in ISS-III; *p* = 0.004) (Figure 3A). In PB, Tregs were found increased in ISS-III, due to the apparent prevalence of resting Tregs (median: 7.0% in ISS-I vs. 14.1% in ISS-II vs. 22.8% in ISS-III; *p* < 0.001), whereas the early-stage MDSCs (eMDSCs) were significantly increased in the low-risk ISS-I group (median: 0.9% in ISS-I vs. 0.1% in ISS-II vs. 0.04% in ISS-III; *p* < 0.001), both contributing to differential immune-suppressive signatures between the three stages (Figure 4A). 

Similarly, the comparison between the distinct cytogenetic groups revealed an uneven distribution for some subsets. Firstly, despite the relative high variance of values among patients, the percentage of B cells was found significantly decreased in the HR group both in the BM and PB (median values in BM: 1.6% in LR vs. 0.9% in HR; *p* = 0.02; median values in PB: 1.4% in LR vs. 0.8% in HR; *p* = 0.04). The total percentage of T cells was also decreased in the BM niche of HR patients (median: 10.7% in LR vs. 7.3% in HR; *p* = 0.007), whereas the prevalence of terminal effector Tregs was 1.6-fold higher in the BM of the HR group (*p* = 0.04) (Figure 3B). PB profiling highlighted differences in the relative frequency of the CD4/CD8 T cell ratio among the CD3+ T cell population, which was partially explained by the significant decrease of the HLA-DR reg CD8+ T cell subset in the HR group (median: 10.9% in LR vs. 6.7% in HR; *p* = 0.01) (Figure 4B).

### 3.4. Immune Signatures May Predict Response to Induction Therapy

Patients’ stratification in well-defined prognostic groups is of utmost need in the clinical practice and especially for highly heterogeneous diseases as MM. The current prognostication systems, based on biochemical measures and the genetic background of APCs, are essential for the clinical management of MM patients; however, they cannot fully predict responses to anti-myeloma treatments. As our cohort was homogenously treated, we evaluated whether distinct basal immune profiling could be of predictive value. 

The differential distribution of several BM subsets in 38 NDMM patients with known responses to VRd induction treatment (12 in CR, 15 in very good partial response (VGPR), 11 in partial response (PR)) revealed significant associations of predictive value. In particular, the most informative markers correlating with therapeutic response were TAMs, erythroblasts, and T cells, especially their CD27+ counterpart. Patients not achieving CR tended to have elevated basal levels of TAMs in their BM (median: 2.3% in CR vs. 3.8% in VGPR vs. 4.4% in PR; *p* = 0.04) and erythroblasts (median: 1.3% in CR vs. 1.3% in VGPR vs. 2.9% in PR; *p* = 0.02), but lower frequencies of CD27+ T cells (median: 76% in CR vs. 71.7% in VGPR vs. 48.6% in PR; *p* = 0.015) (Figure 3C). Of note, the PCA diagram considering the basal levels of these markers could point out a distinct immune signature for those patients achieving CR compared with the pooled profiles of patients’ achieving PR or VGPR (Figure 3D).

The same process was applied in PB in an effort to highlight unique signatures in liquid biopsies that could predict therapeutic outcome. Our analysis revealed distinct Treg profiles among patients with different responses (7 in CR, 17 in VGPR, 16 in PR). In specific, patients who achieved a CR appeared with lower (although not statistically significant) levels of total Tregs, but had significantly higher levels of terminal effector Tregs (median: 21.7% in CR vs. 6.7% in VGPR vs. 3.4% in PR; *p* = 0.008) at the expense of the resting Treg counterpart (median: 4.9% in CR vs. 9.3% in VGPR vs. 20.1% in PR; *p* = 0.007) (Figure 4C). Again, PCA clustering on these variables grouped together patients achieving PR and VGPR, highlighting a clearly unique Treg signature for patients achieving CR (Figure 4D). As mentioned above, the relative frequencies of resting Tregs were noticeably different among the three ISS stages, implying that the effect of this immunosuppressive subset in CR prediction may come from the favorable ISS prognostication. Nevertheless, only 50% of patients achieving CR were of ISS-I stage, thus supporting the independent predictive value of this immune profile in the therapeutic response to induction therapy.

### 3.5. MRD Positivity Is Associated with a Distinct Immune Profile

The evaluation of MRD has emerged as the strongest prognostic factor in MM informing for the depth of response to treatment and has been recently considered as a valuable endpoint to clinical trials and in some cases a critical point for tailored therapeutic strategies [21,22]. Although there are numerous studies highlighting the favorable prognostication of patients achieving MRD negativity [23,24], there is limited information regarding the underlying biology and immune profiling of MRD status. Taking into consideration the pattern of immune distribution revealed by the various multiparametric panels shown herein, we tested for differences in the BM architecture between 20 MRD-negative (MRD−) and 16 MRD-positive (MRD+) patients.

**Figure 5:**
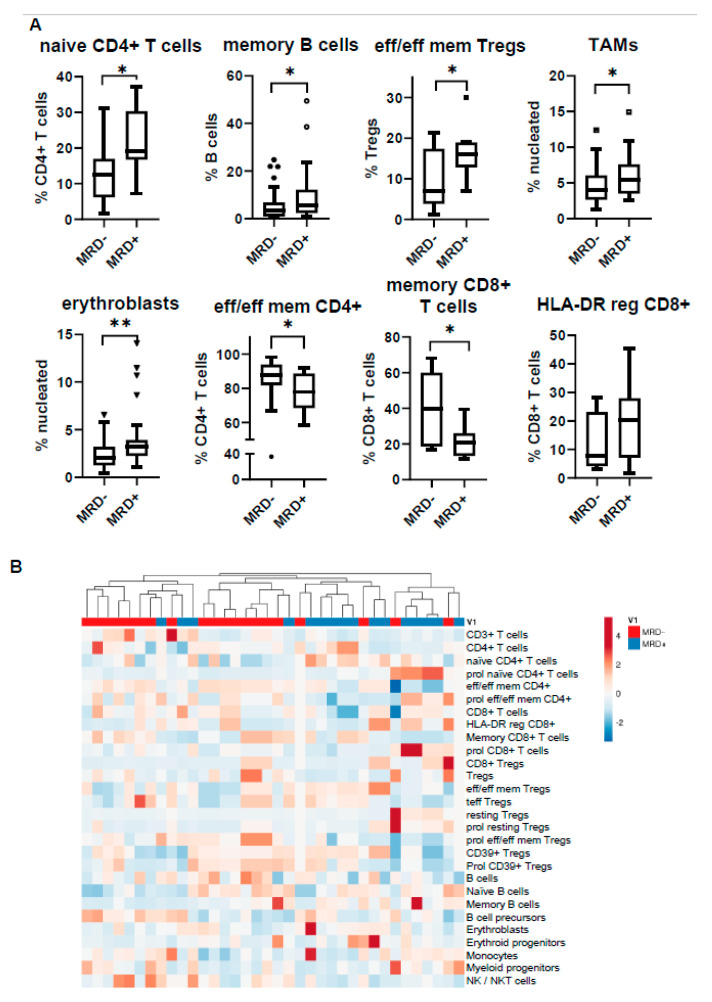
Bone marrow profiling of minimal residual disease (MRD)-positive (MDR+) vs. MRD-negative (MRD−) patients. (**A**) Comparison of subset distribution according to MRD status; (**B**) heatmap displaying the unsupervised hierarchical clustering of MRD patients based on their entire immune composition. * *p* < 0.05, ** *p* < 0.01.

The distribution of the various immune subsets was quite heterogeneous among patients, forming a unique individualized microenvironmental signature for each one of them. Despite the apparent variability, the unsupervised hierarchical model clustered together patients of the same MRD status, highlighting particular differences between MRD− and MRD+ BM cell content (Figure 5). At the unit level, the subsets showing the highest divergence were naïve CD4+ T cells (median: 12,6% in MRD− vs. 19.1% in MRD+ patients; *p* = 0.014), memory B cells (median: 3.3% in MRD− vs. 5.7% in MRD+ patients; *p* = 0.04), effector/effector memory Tregs (median: 7% in MRD− vs. 16% in MRD+; *p* = 0.04), TAMs (median: 4% in MRD− vs. 5.5% in MRD+ patients; *p* = 0.03), and erythroblasts (median: 2% in MRD− vs. 3.2% in MRD+ patients; *p* = 0.006). All the above subpopulations were more abundant in the MRD+ state. On the contrary, the subsets of effector/effector memory CD4+ T cells (median: 87.6% in MRD− vs. 77.9% in MRD+ patients; *p* = 0.04) and memory CD8+ T cells (median: 39.8% in MRD− vs. 20.7% in MRD+ patients; *p* = 0.016) were increased in the MRD– BM microenvironment.

### 3.6. PB Signatures as Indicators for MRD Status

Despite the tremendous advances in current methodologies and their increasing sensitivity levels, at present, liquid biopsy cannot replace BM aspiration for an efficient MRD assessment, due to the minimal—if any—number of circulating clonal cells. Therefore, one of the major challenges in the daily clinical practice is the identification of biomarkers that could accurately depict MRD status via simple and non-invasive testing. We thus tried to examine if any particular immune profile of the various subsets tested could be indicative of the BM-based MRD result.

**Figure 6:**
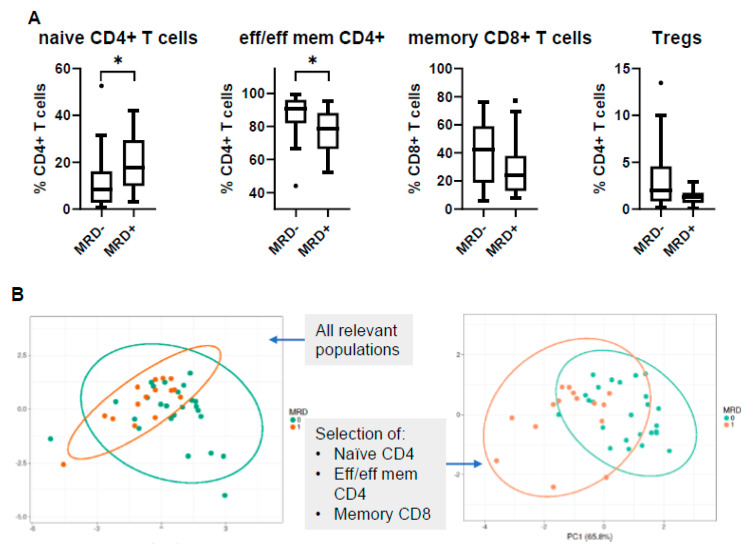

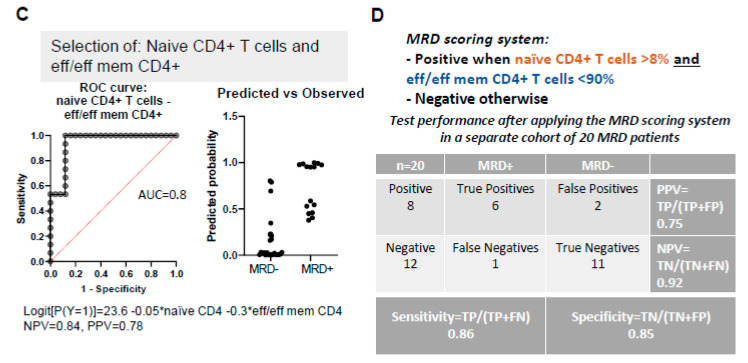
Peripheral blood (PB) profiling of MRD-positive (MRD+) vs. MRD-negative (MRD−) patients. (**A**) Relative frequencies of selected subpopulations according to MRD status; (**B**) PCA analysis including the whole immune pattern as input variables (left); PCA analysis including only selected subsets with significant differences among MRD groups (right); (**C**) selection of naïve CD4+ and effector/effector memory CD4+ T cells allowed the prediction of MRD status with an AUC = 0.8; (**D**) MRD scoring system based on selected cut-off values for naïve CD4+ and effector/effector memory CD4+ T cells, tested on a different patient cohort (*n* = 20). The negative predictive value (NPV) of the scoring system was improved to 0.92. * *p* < 0.05; AUC: area under the curve; TP: true positive; TN: true negative; FP: false positive; FN: false negative; PPV: positive predictive value; NPV: negative predictive value.

As expected, PCA analysis of all tested subsets could not show any significant discrimination between MRD− and MRD+ patients based on PB signature as a whole. Nevertheless, when PCA was performed only with those subsets which individually showed differential distribution between the two groups, the discrimination was quite clear (Figure 6A,B). The most informative immune subsets were naïve CD4+ T cells and effector/effector memory CD4+ T cells, the combination of which conferred an AUC value of 0.8 for a relatively efficient and reliable prediction of the MRD status (Figure 6C). This prediction could be further improved by applying specific cut-off values; a simple MRD scoring system defined by the presence of naïve CD4+ T cells at values higher than 8% and effector/effector memory CD4+ T cells at values lower than 90% could predict MRD-positivity with a satisfactory accuracy, 86% sensitivity, and 85% specificity, when assessed in a separate MM patient cohort (Figure 6D).

## 4. Discussion

Current therapeutic advances together with the emergence of several efficient therapeutic regimens have led to substantial improvement in the clinical management of MM patients, who may now experience extended progression-free periods and prolonged survival [25,26]. Intense research efforts focusing mostly on the molecular features of myeloma cells have shed light in the underlying biology of MM, although thorough understanding of this highly heterogeneous and complex disease remains scanty. The BM microenvironment plays a crucial role during the natural history of MM, and various niche-dynamics have been recognized as an important aspect for disease progression and resistance to therapy [14,15,27]. However, the complexity of the BM composition together with the spatiotemporal altered interplay with myeloma cells [28] restrict the deep comprehension of the mechanisms involved. Contrarily to the majority of relevant reported studies which focus on one immune subset, in the present report, using specifically designed antibody panels, we performed detailed immune profiling of both BM and PB at different disease stages, including at MRD evaluation, in an effort to reveal immune signatures associated with distinct clinical features.

We first looked at differences in the relative frequencies of the major immune subsets into the BM microenvironment of patients at different MM stages. PCL, a rare and aggressive form of plasma cell dyscrasia, showed a unique immune profile, supporting the notion of a distinct entity, which seems to differ not only clinically and genetically from MM [20,29], but also in its relative microenvironmental composition. This notwithstanding, and in agreement with previous reports, our analysis did not highlight any significant differences in the prevalence of lymphocytes, TAMs, or erythroblasts between sMM, newly diagnosed, or treated patients at CR, due to the significant variance of respective measures in each category [30,31]. This divergence among samples highly reflects the dynamic nature of the BM niche, while it also necessitates the identification of clinically relevant immune signatures for more efficient stratification of patients. 

In parallel with the BM profiling, we also applied the same phenotypic analysis in PB-paired samples of the same patients to investigate whether BM microenvironmental features could be echoed in blood circulation. Available studies comparing the relative distribution of individual immune subsets (e.g., Tregs, MDSCs) between BM and PB often lead to contradicting results [31,32,33,34]. However, to the best of our knowledge, this is the first holistic approach comparing the distribution of numerous immune subsets at both sites and at different time-points. Each particular subset identified followed one of the three patterns in BM vs. PB: (i) clear increase/decrease in one site; (ii) similar prevalence in both sites; (iii) no association between sites due to high variability. At diagnosis, significant differences were observed for particular CD4+ and CD8+ T cell subsets, possibly implying different activation or exhaustion levels in PB compared to BM [35]. The relative comparison of monocytes/TAMs showed a higher prevalence in PB, but it is possible that a different selection of phenotypic markers could narrow down these alterations [36]. In line with previous reports, our study did not show any significant differences in the distribution of total Tregs between PB and BM [31,37], although specific subsets with enhanced suppressive functions (i.e., effector/effector memory Tregs, terminal effector Tregs, CD39+ Tregs) prevailed in the BM tumor site. Marsh-Wakefield et al. [37] showed a relative increase of the CD39- Treg compartment in the BM of MM patients, but these findings do not contradict our results, as in our study, only the proliferating CD39+ compartment was substantially higher in the BM and not total CD39+ Tregs. Contrarily to Tregs, MDSCs and particularly the M- and PMN-MDSC compartments tended to have a higher frequency in PB. Nevertheless, besides these phenotypes and the few subsets with similar distribution between BM and PB, the whole immune spectrum analyzed revealed that each site has a unique profile and liquid biopsy could not reflect the BM composition. Moreover, it could be argued that the two sites could be more similar at CR, when tumor burden is decreased. Statistically, fewer subsets revealed significant differences at the CR status, but that was due to the apparent variance of each site, supporting that BM and PB have unique profiles irrespective of disease stage.

The identification of prognostic biomarkers in the NDMM setting is very important for patients’ stratification and subsequent therapeutic management with the most appropriate regimens. The relative distribution of several subsets showed some significant differences among the distinct prognostic groups, probably reflecting a divergent immune status in each category. The most apparent differences between the groups were associated to their respective distribution of T cells and their major CD4+ and CD8+ T cell compartments. Patients with HR aberrations and/or adverse ISS-III stage had relatively lower percentages of total T cells with a skewed increased of CD4/CD8 ratio, which was obvious at both BM and PB (Figure S2). The CD4/CD8 ratio in PB has been reported to decrease during disease progression, and has been considered as an independent unfavorable marker related to advanced disease and increased tumor burden [38,39,40]. Our data verify previous studies and also highlight that the same events take place within the BM. Another difference, apparent in both sites, was the significant decrease of the total B cell compartment in the adverse prognostic groups, finding similar with those of a previous study by Všianská et al. [41]. Finally, a notable finding was the significant increase of NK/NKT cells in the favorable ISS-I stage, probably indicating an advanced innate NK cell-mediated cytotoxicity; besides, previous reports have described numerical and functional impairment of NK effector functions alongside disease continuum [42].

Apart from clinical correlations with established prognostic factors, our findings exceeded the importance of patients’ immune profiling to a clinically relevant level, since unique signatures could significantly relate to different therapeutic responses. Within the BM, a unique profile of NDMM patients characterized by elevated T cells and the CD27+ T cell subset, together with decreased erythroblasts and TAMs could discriminate patients eventually achieving CR to VRd induction treatment. TAMs have lately emerged as a crucial member of the BM microenvironment in MM [43,44], and their elevated numbers—especially of the M2 subtype—have been related to inferior outcomes [45]. We should note that our analysis revealed a skewed ratio towards M2 phenotype in patients with worse responses (statistically non-significant), but the inclusion of this parameter in our PCA models did not improve discrimination compared with the actual total percentage of TAMs. Additionally, the CD27+ T cell compartment comprises cells with unique functions (e.g., immune suppression) and the CD27−/CD27+ ratio has been recently proposed as a marker with independent prognostic value [46].

Similarly, a unique Treg-signature strongly associating with CR was identified in PB. Patients presented with lower percentages of Tregs, but, most importantly, with an increased ratio of terminal effector/resting Tregs responded better to VRd. The clinical impact of Tregs in MM progression has not been validated and reported data may often provide opposing results [31,34,47,48]. Even more uncertain is the role of specific Treg subsets in MM outcome, where available data is sparse. Nevertheless, in a recent study by Kotsakis et al., the authors concluded that higher proportion of circulating terminal effector Tregs with concurrent elimination of the resting Treg subset correlated with an improved clinical response in small and non-small cell lung cancer patients [49].

Following the same strategy described for NDMM, we examined if a specific immune profiling could correlate with MRD status. MRD negativity detected by utilizing highly sensitive techniques (i.e., NGF, NGS) has emerged as the strongest independent prognostic factor, correlating with prolonged progression-free periods and overall survival of MM patients [50,51]. However, the identification of particular immune dissimilarities according to MRD status has not been adequately explored. In a previous study by Paiva et al., the authors proposed unique BM signatures correlating with distinct outcomes [52]. In particular, a profile by elevated erythroblasts and B cell precursors and decreased levels of naïve and memory B cells conferred the most inferior outcome, which was independent from patients’ MRD status, thus implying that immune profiling could supplement MRD status for improved risk stratification [22]. Our analysis revealed a distinct immune profile between MRD+ and MRD– patients, with the latter showing a more experienced adaptive immunity (i.e., CD4+ and CD8+ T cells) phenotype, probably indicative of competent immune surveillance keeping myeloma burden in repression. On the contrary, MRD+ patients were characterized by increased levels of naïve T cells, TAMs and erythroblasts, verifying similar findings from our previous analysis on an independent cohort [53].

Similarly, PB profiling revealed some particular differences between the two MRD states. Though the differences were not as clear as in the BM, instead, MRD-negativity was associated with a shift towards an effector phenotype of CD4+ T cells in concordance with the BM findings. Notably, when applying a simple scoring system based on the levels of naïve CD4+ and effector/effector memory CD4+ T cells, the prediction of a MRD– status in BM was of 92% accuracy. In a relevant approach, Bhutani et al. reported significantly lower numbers of NK cells and an exhausted T cell signature in the PB of MRD+ patients [54].

In conclusion, our results indicate that the immune microenvironment of MM is dynamic and displays unique immune signatures in distinct prognostic groups and disease stages. Most importantly, our findings highlight predictive immune profiles towards both therapeutic response and MRD status. Even though more data is needed to empower these findings, it seems that the analysis of an individualized/personalized immune profiling has strong potential to actively guide therapeutic interventions.

## 5. Conclusions

There is a constant and dynamic interplay between myeloma cells and their microenvironmental cell subsets which plays a crucial role during the course of the disease. Each patient has a unique immune status and particular immune signatures (in both BM and PB) show strong correlations with distinct responses to administered therapy and MRD status. Therefore, the evaluation of each patient’s characteristic and unique immune profile is of clinical relevance and could provide essential information for the more effective individual-based clinical management of MM patients.

## Figures and Tables

**Table 1:** Clinical characteristics of newly diagnosed Multiple Myeloma (MM) patients included in our study.

Clinical Parameters	Patients at Diagnosis (*n* = 53)
Age (years)	66 (44–93)
Male sex (%)	26 (39%)
Hemoglobin (g/dL)	10.8 (8.0–14.7) *
Platelet counts (×10^9^/L)	243 (56–591)
Neutrophil counts/μL	4513 (1000–13,000)
Serum Creatinine (mg/dL)	1.63 (0.5–11.1)
Serum B2MG (mg/L)	5.7 (0.3–26.4)
Serum LDH (U/L)	167 (74–293)
Serum Calcium (mg/dL)	9.6 (8.4–14.4)
BM infiltration (%)	50.7 (0–90)
**ISS stage**	
I	20/53 (38%)
II	18/53 (34%)
III	15/53 (28%)
**FISH aberrations**	
High risk	22/53 (42%)
Low risk	31/53 (58%)
**Heavy chain**	
IgA	13/53 (25%)
IgG	32/53 (60%)
IgD	1/53 (2%)
Light chain only	7/53 (13%)

* All measures in non-categorical parameters show median values with range in parentheses.

**Table 2:** Immune subsets and their corresponding phenotypes analyzed in bone marrow (BM) and peripheral blood (PB) samples.

Immune Subset	Expression of Markers
NGF MRD panel	
Plasma cells	CD38^br^CD138+
B cells	CD19+CD45+
Naïve B cells	CD19+CD27-CD38^-/dim^CD45+SSC^low^
B cell precursors	CD19+CD27-CD38^br^CD45^dim^SSC^low^
Memory B cells	CD19+CD27+CD38^-/dim^CD45+SSC^low^
T cells	CD19-CD45+CD56-SSC^low^
CD27+ T cells	CD19-CD45+CD56-CD27+SSC^low^
NK/NKT cells	CD19-CD45+ CD56-SSC^low^
CD27+ NK/NKT cells	CD19-CD45+CD56-CD27+SSC^low^
Neutrophils	CD45^dim^SSC^high^
Myeloid progenitors	CD38+CD45^dim^CD117+SSC^high^
Monocytes—TAMs	CD38+CD45+CD81+SSC^int^
Mast cells	CD45^dim^CD117^br^
Erythroblasts	CD38-CD45-SSC^low^
Erythroid progenitors	CD38^-/dim^CD45^-/dim^CD117+SSC^low^
**T cell panel**	
T regulatory cells (Tregs)	CD3+CD4+CD25+CD127^low^FoxP3+
Effector/effector memory Tregs (eff/eff mem Tregs)	CD3+CD4+CD25+CD127^low^FoxP3+CD45RA-CD45RO+HLA-DR-CTLA4+
Terminal effector Tregs (teff Tregs)	CD3+CD4+CD25+CD127^low^FoxP3+CD45RA-CD45RO+HLA-DR+ CTLA4+
Resting Tregs	CD3+CD4+CD25+CD127^low^FoxP3+CD45RA-CD45RO+HLA-DR-CTLA4-
CD39+ suppressor Tregs (CD39 Tregs)	CD3+CD4+CD25+CD127^low^FoxP3+CD45RA-CD45RO+CD39+
CD4+ Τ cells	CD3+CD4+
Naïve CD4+ Τ cells	CD3+CD4+CD45RA+CD45RO-
Effector/Effector memory CD4+T cells (eff/eff mem CD4+)	CD3+CD4+CD45RA-CD45RO+
CD8+ T cells	CD3+CD8+
CD8+ Tregs	CD3+CD8+CD25+FoxP3+
Memory CD8+ T cells	CD3+CD8+CD45RO+
HLA-DR regulatory CD8+ T cells (HLA-DR reg CD8+)	CD3+CD8+HLA-DR+
**MDSC panel**	
Polymorphonuclear myeloid-derived suppressor cells (PMN-MDSCs)	CD14-CD11b+CD15+SSC^high^
Early myeloid-derived suppressor cells (eMDSCs)	Lin(CD3/CD14/CD15/CD19/CD56)-HLA-DR-CD33+
Monocytic myeloid-derived suppressor cells (M-MDSCs)	CD11b-CD14+HLA-DR^low/-^CD15-
M1 monocytes	Lin(CD3/CD14/CD15/CD19/CD56)-CD14+CD124-
M2 monocytes	Lin(CD3/CD14/CD15/CD19/CD56)-CD14+CD124+

Abbreviations: br: bright expression; int: intermediate expression; SSC: side scatter.

## Supplementary Materials

The following are available online at https://www.mdpi.com/2072-6694/12/11/3245/s1, Figure S1: (A) Gating strategy of panel 1 for T cell and regulatory T cell analysis (B) Gating strategy of panel 2 for T cell and regulatory T cell analysis. (C) Gating strategy of panel 3 for MDSC analysis, Figure S2: Distribution of total T cells, their CD4+ and CD8+ counterparts, the CD4/CD8 ratio, and NK/NKT frequency in the bone marrow (BM) and peripheral blood (PB) of MM patients at the time of diagnosis, Table S1: Clones and relative providers of antibodies used in the study, Table S2: Relative distribution of various immune subsets in paired bone marrow (BM) and peripheral blood (PB) samples.
