# Supplementary material for: Deep Phenotyping Reveals Distinct Immune Signatures Correlating with Prognostication, Treatment Responses, and MRD Status in Multiple Myeloma
Source: Cancers (Basel). 2020 Nov 4;12(11):3245. doi: 10.3390/cancers12113245 (PMC7692501; doi:10.3390/cancers12113245)
Supplement: Supplementary file 1 [file cancers-12-03245-s001.pdf]

# Supplementary Materials:

# Deep Phenotyping Reveals Distinct Immune Signatures Correlating with Prognostication, Treatment Responses, and MRD Status in Multiple Myeloma

**Konstantinos Papadimitriou, Nikolaos Tsakirakis, Panagiotis Malandrakis, Panagiotis Vitsos, Andreas Metousis, Nikolaos Orologas-Stavrou, Ioannis Ntanasis-Stathopoulos, Nikolaos Kanellias, Evangelos Eleutherakis-Papaiakovou, Panagiotis Pothos, Despina Fotiou, Maria Gavriatopoulou, Efstathios Kastritis, Meletios-Athanasios Dimopoulos, Evangelos Terpos, Ourania E. Tsitsilonis and Ioannis V. Kostopoulos**

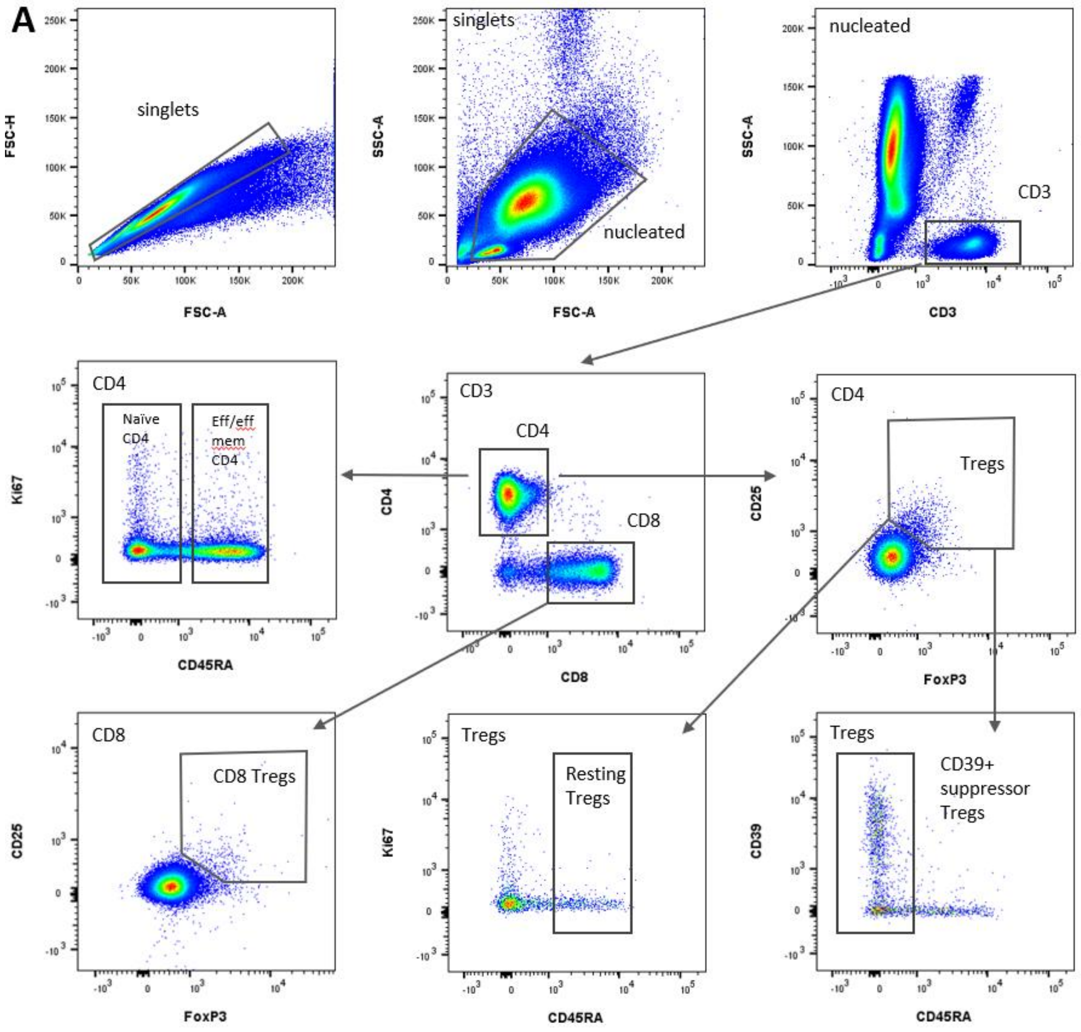

B

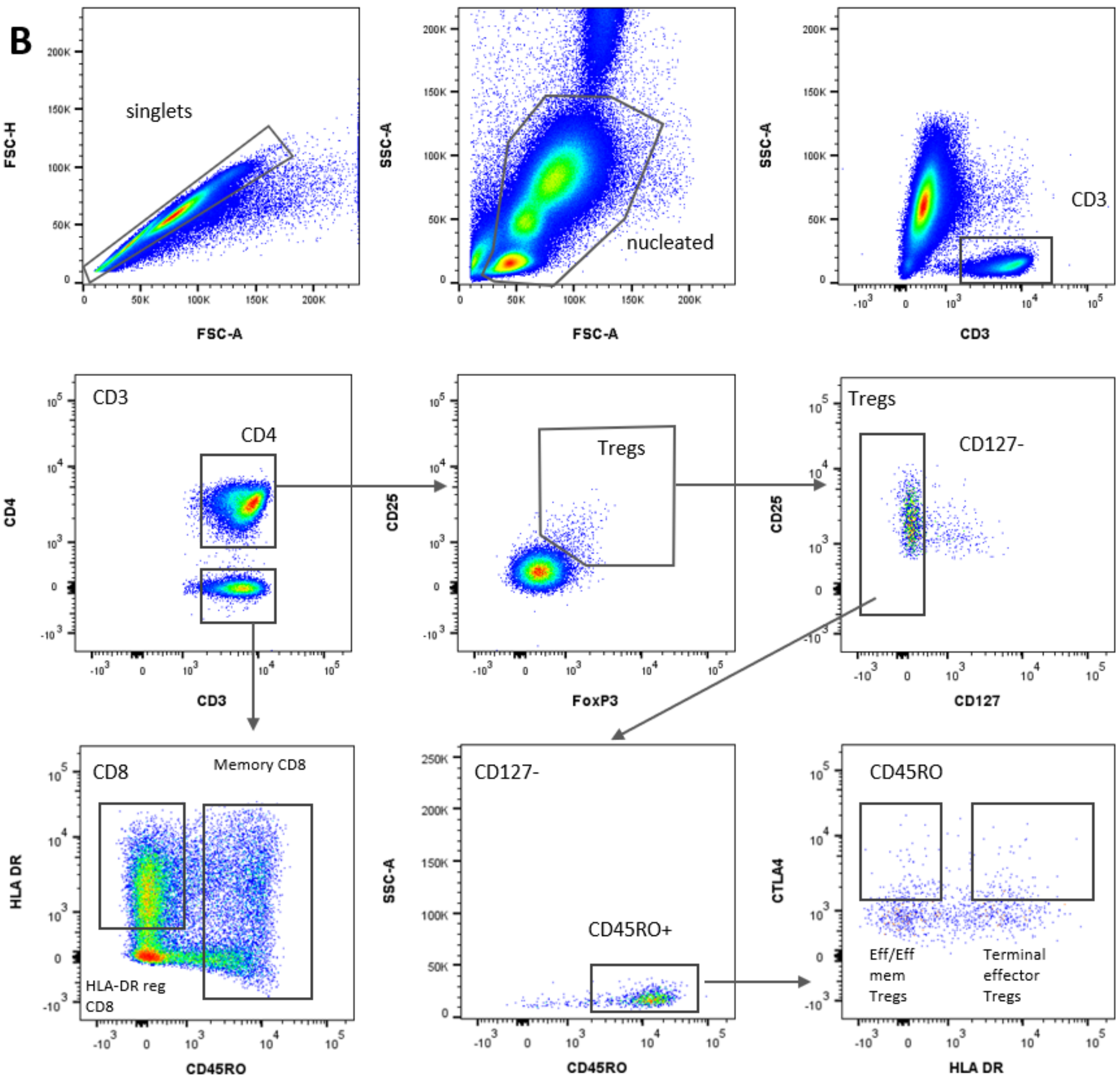

singlets
FSC-H
FSC-A
SSC-A
nucleated
CD3
CD4
CD25
Tregs
FoxP3
CD127-
CD127
CD8
Memory CD8
HLA DR
HLA-DR reg CD8
CD45RO
CD45RO+
CTLA4
Eff/Eff mem Tregs
Terminal effector Tregs

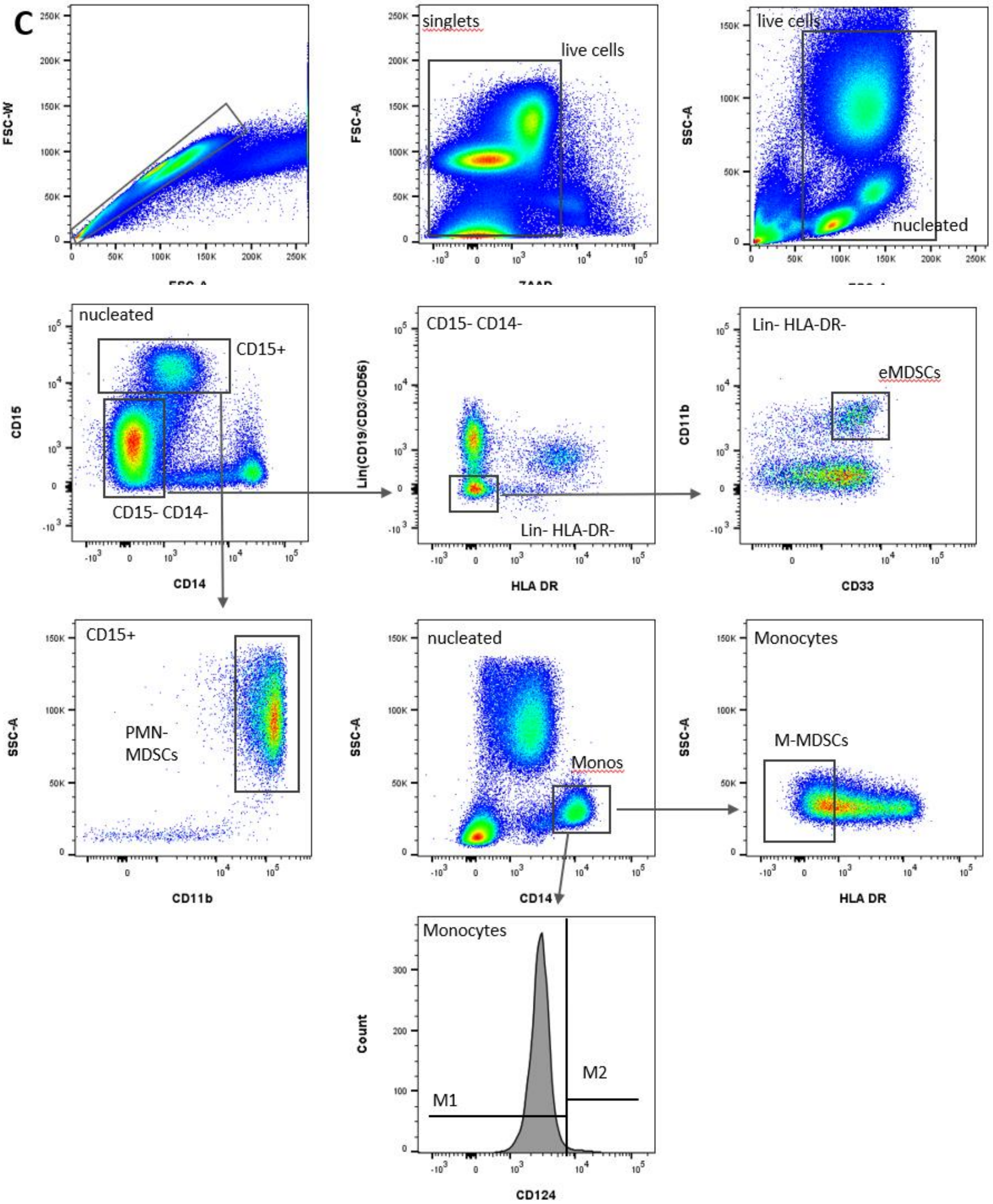


**Figure S1.** (**A**) Gating strategy of panel 1 for T cells and regulatory T cells analysis (**B**) Gating strategy of panel 2 for T cells and regulatory T cells analysis. (**C**) Gating strategy of panel 3 for MDSCs analysis. Live PBMCs were initially selected. Nucleated cells in a CD14—CD15 dot plot were gated for further discrimination of PMN-MDSCs and eMDSCs. The gating strategy followed the recommendations for MDSC characterization [1].

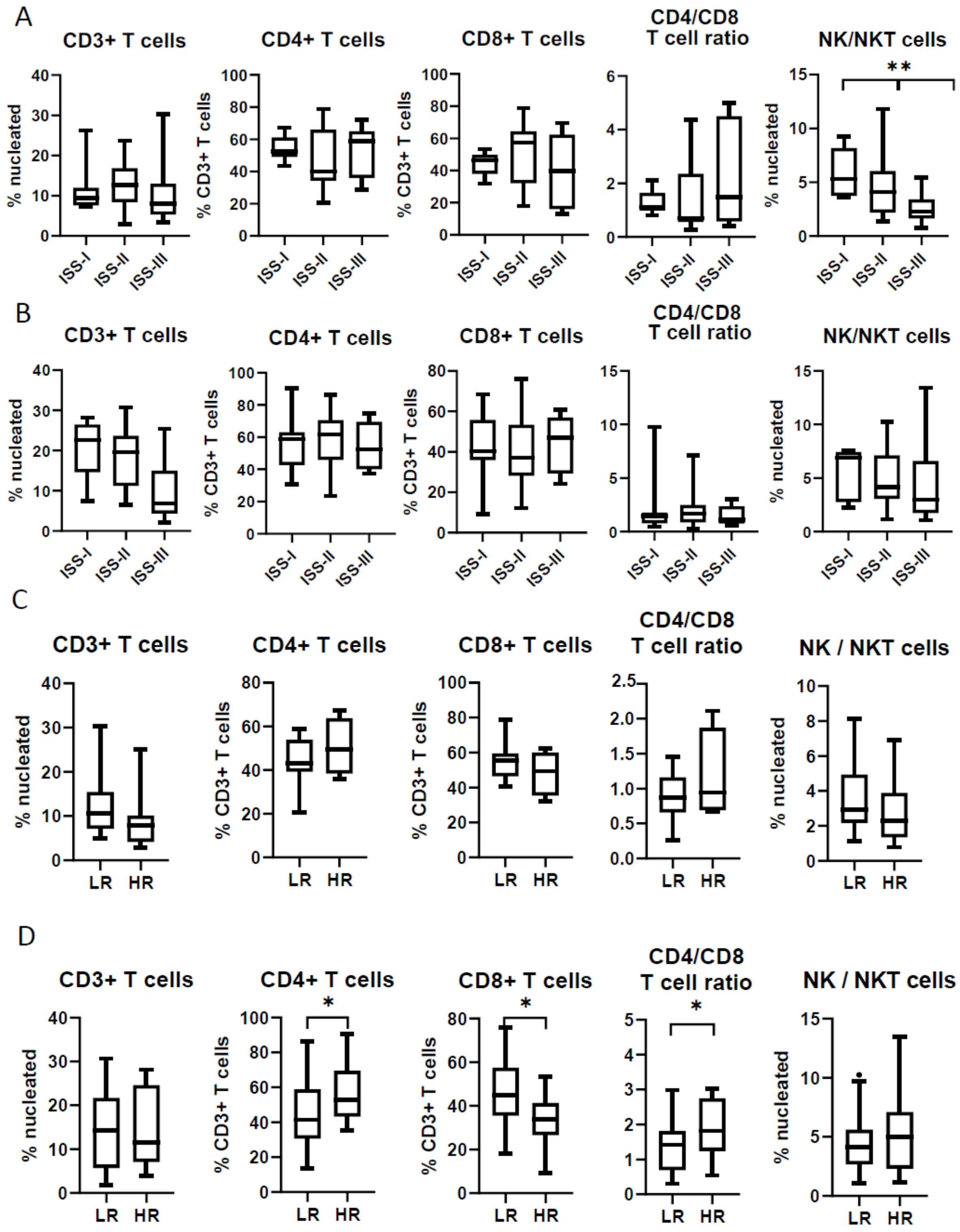


**Figure S2.** Distribution of total T cells, their CD4+ and CD8+ counterpart, the CD4/CD8 ratio and NK/NKT frequency in the bone marrow (BM) and peripheral blood (PB) of MM patients at the time of diagnosis. (**A**) Distribution in BM according to the International Staging System (ISS); (**B**) distribution in PB according to ISS stage; (**C**) distribution in BM according to cytogenetics; (**D**) distribution in PB according to cytogenetics. * $p < 0.05$; ** $p < 0.01$; HR: high-risk; LR: low-risk.

**Table S1:** Clones and relative providers of antibodies used in the study.

| Antibody | Clone | Provider |
|---|---|---|
| CD38-FITC | Multi-epitope | Cytognos, Salamanca, Spain |

| | | |
|---|---|---|
| CD56-PE | C5.9 | Cytognos, Salamanca, Spain |
| CD45-PerCPCy5.5 | HI30 | BD Bioscience, San Jose, CA, USA |
| CD138-BV421 | MI15 | BD Bioscience, San Jose, CA, USA |
| CD27-BV510 | O323 | BD Bioscience, San Jose, CA, USA |
| CD19-PC7 | J3-119 | Beckman Coulter, Brea, CA, USA |
| CD117-APC | 104D2 | BD Bioscience, San Jose, CA, USA |
| CD81-APCC750 | M38 | Cytognos, Salamanca, Spain |
| Kappa-APC | Polyclonal | Agilent, Santa Clara, CA, USA |
| Lambda-APCC750 | Polyclonal | Cytognos, Salamanca, Spain |
| CD3-FITC | HIT3a | BD Bioscience, San Jose, CA, USA |
| CD4-APC-Cy7 | RPA-T4 | BD Bioscience, San Jose, CA, USA |
| CD8-PerCPCy5.5 | SK1 | BD Bioscience, San Jose, CA, USA |
| CD25-APC | 555434 | BD Bioscience, San Jose, CA, USA |
| FoxP3-PE | 236A/E7 | BD Bioscience, San Jose, CA, USA |
| CD127-BV510 | HIL-7R-M21 | BD Bioscience, San Jose, CA, USA |
| CD39-BV421 | TU66 | BD Bioscience, San Jose, CA, USA |
| Ki67-BV510 | B56 | BD Bioscience, San Jose, CA, USA |
| CD45RA-PC7 | HI100 | BD Bioscience, San Jose, CA, USA |
| CD45RO-PerCPCy5.5 | UCHL1 | BD Bioscience, San Jose, CA, USA |
| CTLA4-BV421 | BNI3 | BD Bioscience, San Jose, CA, USA |
| HLA-DR-PC7 | G46-6 | BD Bioscience, San Jose, CA, USA |
| CD14-FITC | M5E2 | BD Bioscience, San Jose, CA, USA |
| CD11b-PE | Mac-1 | BD Bioscience, San Jose, CA, USA |
| 7-AAD | | BD Bioscience, San Jose, CA, USA |
| CD124-BV421 | hIL4R-M57 | BD Bioscience, San Jose, CA, USA |
| CD33-BV510 | WM53 | BD Bioscience, San Jose, CA, USA |
| HLA-DR-PE | TU36 | BD Bioscience, San Jose, CA, USA |
| CD15-APC | HI98 | BD Bioscience, San Jose, CA, USA |
| CD3-APC-Cy7 | SK7 | BD Bioscience, San Jose, CA, USA |
| CD19-APC-Cy7 | 557791 | BD Bioscience, San Jose, CA, USA |
| CD56-APC-Cy7 | HCD56 | Biolegend, San Diego, CA, USA |

**Table S2.** Relative distribution of various immune subsets in paired bone marrow (BM) and peripheral blood (PB) samples.

| Immune subset | At diagnosis | | *p* value | In complete remission | | *p* value |
|---|---|---|---|---|---|---|
| | BM | PB | | BM | PB | |
| T cells<br>(% of nucleated cells) | 10.52 ± 6.52 | 13.57 ± 8.09 | **0.004*** | 8.64 ± 7.30 | 11.25 ± 9.85 | 0.85 |
| CD27+ T cells<br>(% of T cells) | 69.80 ± 20.41 | 67.31 ± 20.29 | 0.26 | 50.52 ± 19.12 | 47.58 ± 26.41 | 0.74 |
| CD4+ T cells<br>(% of CD3+ cells) | 51.10 ± 14.70 | 61.40 ± 15.7 | **<0.001** | 40.94 ± 16.06 | 46.33 ± 18.2 | **<0.001** |
| Naïve CD4+ T cells<br>(% of CD4+cells) | 28.43 ± 21.16 | 34.3 ± 21.5 | **0.002** | 15.48 ± 14.02 | 14.50 ± 12.81 | 0.39 |
| Proliferating naïve CD4+ T cells<br>(% of CD4+ cells) | 0.59 ± 0.72 | 0.90 ± 0.71 | **0.014** | 0.34 ± 0.45 | 0.21 ± 0.33 | **0.006** |

| | | | | | | |
|---|---|---|---|---|---|---|
| Effector / effector memory CD4+ T cells (% of CD4+ cells) | 68.44 ± 21.47 | 62.13 ± 21.70 | **<0.001** | 82.79 ± 14.84 | 83.89 ± 13.5 | 0.36 |
| Proliferating Effector/effector memory CD4+ T cells (% of CD4+ cells) | 1.55 ± 0.85 | 1.94 ± 0.90 | 0.06 | 2.04 ± 0.91 | 1.9 ± 0.88 | 0.34 |
| Tregs (% of CD4+ cells) | 1.19 ± 0.86 | 1.10 ± 0.80 | 0.57 | 1.74 ± 1.48 | 1.71 ± 1.52 | 0.88 |
| Resting Tregs (% of Tregs) | 15.33 ± 16.44 | 16.30 ± 19.75 | 0.92 | 8.11 ± 19.24 | 9.03 ± 19.21 | 0.51 |
| Proliferating resting Tregs (% of Tregs) | 3.11 ± 4.69 | 6.25 ± 10.54 | 0.26 | 1.16 ± 1.98 | 0.79 ± 1.79 | 0.54 |
| Effector/effector memory Tregs (% of Tregs) | 14.60 ± 8.23 | 9.33 ± 8.03 | **0.03** | 12.18 ± 7.83 | 12.06 ± 11.42 | 0.51 |
| Proliferating effector/effector memory Tregs (% of Tregs) | 0.15 ± 0.09 | 1.16 ± 0.62 | 0.43 | 1.48 ± 0.43 | 1.41 ± 1.32 | 0.74 |
| Terminal effector Tregs (% of Tregs) | 16.01 ± 10.9 | 7.43 ± 6.5 | **0.01** | 14.62 ± 12.15 | 14.06 ± 10.4 | 0.59 |
| CD39+ suppressor Tregs (% of Tregs) | 36.13 ± 31.89 | 47.2 ± 22.2 | 0.14 | 48.12 ± 30 | 50.94 ± 29.46 | 0.32 |
| Proliferating CD39+ suppressor Tregs (% of Tregs) | 4.54 ± 6.28 | 8.1 ± 6.3 | **0.003** | 6.19 ± 4.66 | 6.89 ± 4.66 | 0.41 |
| CD8+ T cells (% of CD3+ cells) | 46.50 ± 15.06 | 36.8 ± 15.9 | **<0.001** | 53.14 ± 15.91 | 43.82 ± 18.04 | **<0.001** |
| Proliferating CD8+ T cells (% of CD8 cells) | 2.17 ± 1.23 | 2.52 ± 1.73 | 0.27 | 1.74 ± 1.19 | 1.61 ± 1.13 | 0.39 |
| HLA-DR regulatory CD8+ T cells (% of CD8 cells) | 11.93 ± 7.65 | 7.30 ± 5.74 | **<0.001** | 16.72 ± 14.34 | 17.66 ± 17.65 | 0.93 |
| Memory CD8+ T cells (% of CD8 cells) | 27.48 ± 15.41 | 27.1 ± 13.8 | 0.87 | 34.56 ± 19.97 | 31.79 ± 20.90 | 0.22 |
| CD8+ Tregs (% CD8+ cells) | 0.25 ± 0.82 | 0.29 ± 1.02 | 0.28 | 0.34 ± 1.39 | 0.27 ± 1.17 | 0.27 |
| B cells (% of nucleated cells) | 1.49 ± 1.15 | 1.42 ± 0.93 | 0.69 | 3.51 ± 4.34 | 4.2 ± 5.24 | 0.54 |
| Memory B cells (% of B cells) | 34.21 ± 21.7 | 36.90 ± 20.73 | **0.03** | 9.21 ± 15.28 | 10.34 ± 12.47 | 0.67 |
| Naïve B cells (% of B cells) | 46.74 ± 21.79 | 61.61 ± 20.56 | **<0.001** | 48.73 ± 25.18 | 63.47 ± 25.14 | 0.74 |
| B cell precursors (% of B cells) | 19.09 ± 16.54 | 2.02 ± 2.11 | **<0.001** | 37.17 ± 27.07 | 1.07 ± 1.25 | **<0.001** |
| PMN-MDSCs (% of mononuclear cells) | 2.38 ± 3.53 | 9.86 ± 11.82 | **0.03** | 3.85 ± 5.02 | 14.18 ± 13.31 | 0.07 |
| M-MDSCs (% of mononuclear cells) | 6.12 ± 4.02 | 10.11 ± 5.38 | **0.04** | 11.92 ± 10.81 | 7.64 ± 10.21 | 0.58 |
| eMDSCs | 0.32 ± 0.49 | 0.55 ± 1.01 | 0.32 | 0.51 ± 0.65 | 0.85 ± 0.80 | 0.36 |

| | | | | | | |
|---|---|---|---|---|---|---|
| (% of mononuclear cells) | | | | | | |
| Monocytes PB / TAMS BM (% of nucleated cells) | 3.16 ± 1.67 | 6.63 ± 2.63 | **<0.001** | 5.24 ± 2.91 | 6.87 ± 4.41 | 0.12 |
| M1 monocytes (% of monocytes) | 97.8 ± 0.94 | 98.26 ± 0.64 | 0.10 | 98.11 ± 1.24 | 97.27 ± 1.49 | 0.84 |
| M2 monocytes (% of monocytes) | 2.20 ± 0.94 | 1.72 ± 0.64 | 0.09 | 1.89 ± 1.24 | 2.73 ± 1.49 | 0.06 |
| NK/NKT cells (% nucleated cells) | 3.23 ± 1.84 | 4.43 ± 2.73 | **0.005** | 3.02 ± 2.76 | 3.94 ± 3.14 | 0.08 |
| CD27+ NK/NKT cells (% of NK/NKT cells) | 17.01 ± 10.23 | 14.20 ± 9.91 | **0.016** | 13.6 4 ±7.15 | 15.24 ± 8.32 | 0.12 |
| Neutrophils (% nucleated cells) | 68.11 ± 11.99 | 65.19 ± 13.04 | 0.55 | 60.88 ± 23.66 | 63.88 ± 19.23 | 0.24 |

* *p* values in bold characters denote statistically significant differences.

## Reference

1. Bronte, V.; Brandau, S.; Chen, S.-H.; Colombo, M.P.; Frey, A.B.; Greten, T.F.; Mandruzzato, S.; Murray, P.J.; Ochoa, A.; Ostrand-Rosenberg, S.; et al. Recommendations for myeloid-derived suppressor cell nomenclature and characterization standards. *Nat. Commun.* **2016**, *7*, 12150, doi:10.1038/ncomms12150.
